# Lower Myeloperoxidase-ANCA Titres at Diagnosis Are Associated with End-Stage Kidney Disease Progression During Follow-Up in Rituximab-Treated Patients with Microscopic Polyangiitis

**DOI:** 10.3390/medicina61111892

**Published:** 2025-10-22

**Authors:** Oh Chan Kwon, Jang Woo Ha, Yong-Beom Park, Sang-Won Lee

**Affiliations:** 1 Division of Rheumatology, Department of Internal Medicine, Gangnam Severance Hospital, Yonsei University College of Medicine, Seoul 06273, Republic of Korea; ockwon@yuhs.ac; 2 Division of Rheumatology, Department of Internal Medicine, Yongin Severance Hospital, Yonsei University College of Medicine, Yongin 16995, Gyeonggi-do, Republic of Korea; hjwnmk@yuhs.ac; 3 Division of Rheumatology, Department of Internal Medicine, Yonsei University College of Medicine, Seoul 03722, Republic of Korea; 4 Institute for Immunology and Immunological Diseases, Yonsei University College of Medicine, Seoul 03722, Republic of Korea

**Keywords:** antineutrophil cytoplasmic antibody, microscopic polyangiitis, myeloperoxidase, end-stage kidney disease, rituximab

## Abstract

*Background and Objectives*: To investigate whether myeloperoxidase (MPO)-antineutrophil cytoplasmic antibody (ANCA) titres at diagnosis are associated with the risk of end-stage kidney disease (ESKD) progression in patients with microscopic polyangiitis (MPA) treated with rituximab. *Materials and Methods*: This retrospective cohort study included 34 patients with MPA who received rituximab. Clinical data, including MPO-ANCA titres at diagnosis and ESKD progression during follow-up, were assessed. Receiver operating characteristic (ROC) curve analysis was performed to assess whether MPO-ANCA titres could predict ESKD progression. The optimal cut-off value of MPO-ANCA titres was determined where the sum of sensitivity and specificity was at a maximum. Based on this cut-off value, patients were categorised into two groups, and the relative risk (RR) of ESKD progression was estimated. *Results*: During a median follow-up of 39.5 months, seven patients (20.6%) progressed to ESKD. ROC curve analysis showed a significant inverse association between MPO-ANCA titres and ESKD progression (AUC 0.254, 95% confidence interval [CI] 0.046, 0.462 *p* = 0.048). The optimal cut-off of MPO-ANCA titres was 81.0 IU/mL, which yielded a sensitivity and specificity of 70.4% and 85.7%, respectively. The RR of ESKD progression was significantly higher in those with MPO-ANCA titres ≤ 81.0 IU/mL than in those with MPO-ANCA titres > 81.0 IU/mL (42.9% vs. 5.0%, RR 14.250, 95% CI 1.469, 138.271). *Conclusions*: Lower MPO-ANCA titres at diagnosis may be associated with a higher risk of ESKD progression in rituximab-treated MPA patients. These findings suggest that MPO-ANCA titres may be useful in guiding therapeutic decisions for MPA.

## 1. Introduction

Microscopic polyangiitis (MPA) is a subtype of antineutrophil cytoplasmic antibody (ANCA)-associated vasculitis (AAV) characterised by fibrinoid necrotising inflammation with no or few immune complex depositions in small-sized vessels [1]. MPA primarily involves the lungs and kidneys, leading to corresponding clinical manifestations, such as pulmonary fibrosis, interstitial lung disease, and pauci-immune glomerulonephritis (GN), and may provoke diffuse alveolar haemorrhage and end-stage kidney disease (ESKD) in severe cases [2,3]. Rituximab, which targets CD20-positive B cells, plays a fundamental role in the treatment of MPA as an induction and maintenance therapy [4,5]. Given the role of circulating myeloperoxidase (MPO)-ANCA in the initiation and aggravation of MPA [6], depleting B cells producing MPO-ANCA might be critical for the treatment of MPA. Therefore, it could be reasonably speculated that the efficacy of rituximab in preventing the occurrence of poor outcomes of MPA, particularly progression to ESKD during follow-up, might be greater in MPA patients having a higher titre of MPO-ANCA. Conversely, it could be assumed that rituximab might not be sufficiently efficient for the treatment of MPA among patients having a relatively low circulating MPO-ANCA titres [4,5,6]. Therefore, we hypothesised that a initial lower MPO-ANCA titre at diagnosis may be associated with poor outcomes during follow-up in rituximab-treated MPO-ANCA-positive MPA patients. However, to date, few studies have investigated whether the initial MPO-ANCA titres can estimate the frequency of ESKD progression during follow-up in rituximab-treated MPA patients. Hence, in this study, we investigated whether MPO-ANCA titres at diagnosis are associated with ESKD progression during follow-up in rituximab-treated MPA patients.

## 2. Materials and Methods

### 2.1. Study Patients

**Figure 1:**
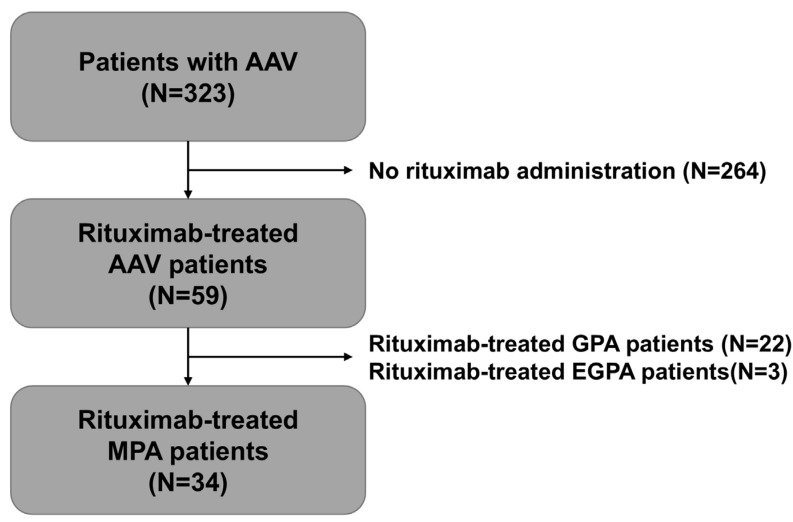
Patients’ selection. AAV: ANCA-associated vasculitis; ANCA: antineutrophil cytoplasmic antibody; GPA: granulomatosis with polyangiitis; EGPA: eosinophilic granulomatosis with polyangiitis; MPA: microscopic polyangiitis.

We retrospectively reviewed the medical records of the 323 patients with AAV who were enrolled and included in the observational cohort of Korean AAV patients and selected the study subjects according to the inclusion criteria: (i) first classification at this hospital; (ii) fulfilment of the 2007 algorithm for AAV, 2012 nomenclature of vasculitis, and 2022 new criteria for MPA [1,2,3]; (iii) sufficiently detailed clinical data, especially regarding MPO-ANCA titre at diagnosis and ESKD progression during follow-up; (iv) a follow-up duration of >6 months after diagnosis; (v) no medical history of significant chronic kidney disease before diagnosis; (vi) no concomitant malignancies or serious infectious diseases mimicking MPA [3]; (vii) no previous exposure to immunosuppressive drugs or other drugs affecting kidney function before diagnosis; (viii) presence of MPO-ANCA (or perinuclear [P]-ANCA) at diagnosis; (ix) presence of evidence supporting renal vasculitis [2]; and (x) administration of rituximab as an induction therapeutic regimen after diagnosis. Among the 323 patients with AAV, 264 patients with AAV were excluded because rituximab was not administered, and of these excluded patients, 167 had ever received cyclophosphamide as the remission induction therapeutic regimen. Further, among the 59 rituximab-treated AAV patients, 22 GPA and 3 EGPA patients were excluded. Finally, 34 rituximab-treated MPA patients were analysed in this study (Figure 1).

### 2.2. Ethical Statement

This study was approved by the Institutional Review Board (IRB) of Severance Hospital, Seoul, Republic of Korea (IRB No. 4-2020-1071), and was conducted in accordance with the Declaration of Helsinki. Given the retrospective design of the study and use of anonymised patient data, the requirement for written informed consent was waived.

### 2.3. Clinical Data at AAV Diagnosis

This study collected age, sex, body mass index (BMI), and smoking history (current or ex-smoker) at the time of MPA diagnosis as baseline demographic information. According to the 2022 classification criteria for MPA proposed by a joint group of the American College of Rheumatology and the European Alliance of Associations for Rheumatology, both MPO-ANCA/proteinase 3 (PR3)-ANCA positivity and P-ANCA/cytoplasmic (C)-ANCA positivity were accepted as ANCA positive [3,7]. The Birmingham vasculitis activity score (BVAS) and the five-factor score (FFS) at diagnosis were collected [8,9], and laboratory results at diagnosis, particularly the erythrocyte sedimentation rate (ESR) and C-reactive protein (CRP) levels, were recorded. ESKD progression during follow-up was evaluated as a poor outcome of MPA, and in this study, we defined ESKD as the medical condition that requires renal replacement therapy [10]. In this study, we arbitrarily applied different concepts for the follow-up duration based on ESKD for the two groups. For the ESKD group, we determined it as the period from MPA diagnosis to the diagnosis of ESKD, whereas for the non-ESKD group, we defined it as the period from MPA diagnosis to the last visit to the vasculitis clinic. In addition, along with rituximab, glucocorticoids and immunosuppressive drugs administered during follow-up were reviewed.

### 2.4. Statistical Analyses

We conducted all statistical analyses using SPSS software (version 28.0; IBM Corporation, Chicago, IL, USA). We expressed continuous variables as median values with 25 to 75 percentiles, whereas categorical variables were expressed as the number of cases with percentages. We calculated the correlation coefficient (r) between the two continuous variables using the Pearson correlation analysis. We used a receiver operating characteristic (ROC) curve to obtain the area under the curve and confirm its significance. In particular, we determined the cut-off for progression to ESKD using the ROC curve by selecting the maximum sum of sensitivity and specificity. We assessed the relative risk (RR) of the cut-off for progression to ESKD using the contingency tables with the chi-square test. Finally, we considered a *p*-value less than 0.05 to be statistically significant.

## 3. Results

### 3.1. Characteristics of Study Subjects

Regarding variables at diagnosis, the median age of the 34 rituximab-treated MPA patients was 59.0 years, and 29.4% of the patients were male. MPO-ANCA (or P-ANCA) was positive in all patients, and the median MPO-ANCA titre was 104.0 IU/mL. The median BVAS, FFS, ESR, and CRP levels were 15.5, 2.0, 80.0 mm/h, and 9.3 mg/L, respectively. In terms of variables related to kidney function, the median blood urea nitrogen, serum creatinine, serum albumin, and random urine protein/creatinine ratio were 22.2 mg/dL, 1.4 mg/dL, 3.4 g/dL, and 1.1, respectively. Additionally, antinuclear antibody, anti-Ro, and anti-La were detected in six, two, and one patients, respectively. However, none were classified as having autoimmune connective tissue diseases. Regarding variables during follow-up, 7 of the 34 patients progressed to ESKD, with a median follow-up duration based on ESKD of 39.5 months. During the remission induction therapeutic regimens, rituximab was administered to all 34 patients according to the inclusion criteria. Further, 34 patients received glucocorticoids along with rituximab treatment and during remission maintenance therapy. During the remission maintenance therapeutic regimens, azathioprine and mycophenolate mofetil were administered to 64.7% and 55.9% of patients, respectively. Of the 34 patients, 8 and 5 received tacrolimus and methotrexate, respectively (Table 1).

### 3.2. Correlation Analysis

MPO-ANCA titres did not significantly correlate with BVAS (r = 0.163), FFS (r = 0.101), or CRP (r = 0.182). MPO-ANCA titres tended to correlate with ESR (R = 0.340, *p* = 0.053); however, this was not statistically significant. Additionally, MPO-ANCA titres were not significantly correlated with blood urea nitrogen, serum creatinine, serum albumin, and random urine protein/creatinine ratio (Table S1).

### 3.3. Cut-Off and Relative Risk for ESKD

**Figure 2:**
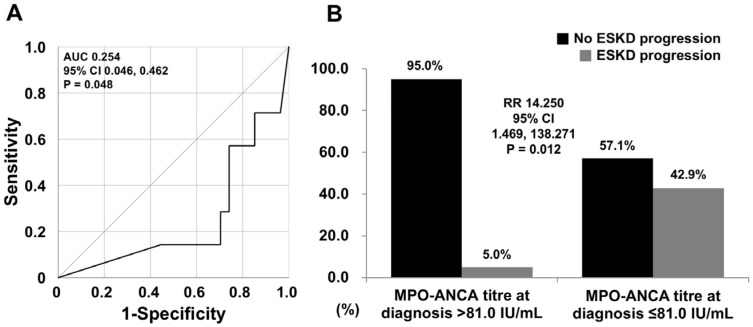
(**A**) Determination of a cut-off of MPO-ANCA titres for ESKD and (**B**) Relative risk of a cut-off of MPO-ANCA titres for ESKD progression. MPO: myeloperoxidase; ANCA: antineutrophil cytoplasmic antibody; ESKD: end-stage kidney disease; AUC: area under the curve; RR: relative risk.

MPO-ANCA titres exhibited a significant ROC curve for ESKD progression (AUC 0.254, 95% confidence interval [CI] 0.046, 0.462, *p* = 0.048); because of the inverse correlation between the two, the curve was presented below the reference line. Additionally, the optimal cut-off of MPO-ANCA titres was determined as 81.0 IU/mL, where the sensitivity and specificity were 70.4% and 85.7%, respectively (Figure 2A). When the 34 patients were divided into two groups according to the cut-off of MPO-ANCA titre as 81.0 IU/mL, the frequency of ESKD progression in patients with MPO-ANCA titre at diagnosis ≤ 81.0 IU/mL was significantly elevated compared to patients with MPO-ANCA titre at diagnosis > 81.0 IU/mL (42.9% vs. 5.0%, *p* = 0.012). Additionally, patients with MPO-ANCA titre at diagnosis ≤ 81.0 IU/mL exhibited a higher risk for ESKD progression than those without (RR 14.250, 95% CI 1.469, 138.271) (Figure 2B).

## 4. Discussion

In this study, we investigated whether MPO-ANCA titres at diagnosis were associated with ESKD progression during follow-up in MPA patients in whom rituximab was administered as the first induction therapeutic regimen. Several interesting findings were obtained. First, MPO-ANCA titres at diagnosis did not correlate with cross-sectional AAV activity, acute-phase reactants, or kidney function at diagnosis in rituximab-treated MPA patients. Second, MPO-ANCA titres at diagnosis exhibited a significant AUC for ESKD progression during follow-up in rituximab-treated MPA patients. Third, among rituximab-treated MPA patients, patients belonging to a lower MPO-ANCA titre group exhibited a higher risk for ESKD progression than those belonging to a higher MPO-ANCA titre group. Therefore, we conclude that the initial MPO-ANCA titres at diagnosis could be useful in predicting the efficacy of rituximab for preventing ESKD progression during follow-up in rituximab-treated MPA patients.

ANCA positivity, particularly MPO-ANCA (or P-ANCA), has been known as a risk factor positively associated with ESKD progression in MPA patients alongside higher chronic kidney disease classes and unfavourable histopathological findings [11,12]. Also, we had demonstrated a significant association between MPO-ANCA (or P-ANCA) positivity at diagnosis and future progression to ESKD among patients enrolled in the same AAV cohort as this study [13]. However, focusing on MPO-ANCA titres, this study yielded a different result: an inverse association between MPO-ANCA titres and ESKD progression during follow-up in MPO-ANCA (or P-ANCA)-positive and rituximab-treated MPA patients.

Moreover, there are several discordances between our previous study and this study. The previous study reported the association between first-year cumulative MPA-ANCA titres and all-cause mortality during the follow-up period in patients with all types of AAV [14]. In contrast, this study examined the association between cross-sectional MPA-ANCA titres measured at diagnosis and progression to ESKD during the follow-up period in rituximab-treated MPO-ANCA-positive MPA patients. Accordingly, there were apparent differences in the study goals and study subjects between the previous and present studies. In addition, in our previous study, the presence of MPO-ANCA (or P-ANCA) at diagnosis was identified as a major risk factor for the deterioration in kidney function during the disease course in MPA patients [15]; however, this analysis could not be performed in the present study because this study included only MPO-ANCA-positive MPA patients, making comparative analysis impossible. Therefore, the background for the initiation of this study was to select only MPA patients with positive MPO-ANCA and determine whether the therapeutic and preventive effect of RTX on progression to ESKD would be better in patients with higher MPO-ANCA titre or with lower MPO-ANCA titre measured at diagnosis.

We made the following hypothesis. Lower MPO-ANCA titres but high BVAS at diagnosis suggest that MPO-ANCA-producing B cells may play a relatively less critical role in the pathophysiology of MPA, while other autoreactive immune cell-mediated inflammatory responses may make a major contribution to the development and exacerbation of MPA [6,16]. Therefore, given that rituximab is a biological agent targeting CD20-positive B cells [17], it can be inferred that patients with lower MPO-ANCA titres at diagnosis may have some trouble in achieving sufficient efficacy of rituximab during the earlier disease course. For these reasons, among patients with lower MPO-ANCA titres, rituximab might have had difficulty in effectively preventing persistent and chronic renal fibrosis, and ultimately could not reduce the frequency of ESKD progression.

Additionally, we attempted to investigate whether lower MPO-ANCA titres at diagnosis could independently predict ESKD progression during the follow-up period based on ESKD among other risk factors using Cox proportional hazards analyses. In univariable Cox analysis, blood urea nitrogen and creatinine at diagnosis (hazard ratio [HR] 1.050, *p* = 0.049, and HR 2.069, *p* = 0.021, respectively) were significantly associated with ESKD progression during follow-up in rituximab-treated MPA patients. Also, fasting glucose at diagnosis (*p* = 0.088) tended to be associated with ESKD progression during follow-up, despite no statistical significance. However, MPO-ANCA titre at diagnosis ≤ 81.0 IU/mL was not significantly associated with future progression to ESKD in rituximab-treated MPA patients.

Nonetheless, given that lower MPO-ANCA titres were significantly correlated with a higher frequency of ESKD progression in the risk-assessing analysis, we decided to include MPO-ANCA titre at diagnosis ≤ 81.0 IU/mL in multivariable Cox analysis along with fasting glucose, blood urea nitrogen, and serum creatinine. In multivariable Cox analysis, only serum creatinine at diagnosis (HR 3.774, 95% confidence interval [CI] 1.034, 13.782) could independently predict ESKD progression during follow-up. However, MPO-ANCA titre at diagnosis ≤ 81.0 IU/mL (*p* = 0.072) was proved not to be independently associated with future progression to ESKD (Table S2).

On the other hand, although age was not included in multivariable Cox analysis described in Table S2, age has also been considered to be a major risk factor for progression to ESKD, comparable to serum creatinine at diagnosis among AAV patients. To further compare the predictive potential among age, initial renal function represented by creatinine levels, and MPO-ANCA titres for an unwanted advance to renal failure, multivariable Cox proportional hazard analysis with these three variables for progression to ESKD was conducted, such as age, serum creatinine levels, and MPO-ANCA titre ≤ 81.0 IU/mL at diagnosis. We found that only serum creatinine at diagnosis (HR 2.650, 95% CI 1.258, 5.582) was independently associated with progression to ESKD during follow-up, but not age (HR 0.963, 95% CI 0.894, 1.037) or MPO-ANCA titre ≤ 81.0 IU/mL (HR 5.542, 95% CI 0.450, 68.302). In the present study, however, it was not the objective of this study to prove that the predictive potential of MPO-ANCA titre for progression to ESKD is comparable or non-inferior to that of conventional risk factors. As a pilot study, we attempted to demonstrate that lower MPO-ANCA titres at diagnosis could be associated with insufficient efficacy of rituximab for preventing progression to ESK in MPO-ANCA-positive MPA patients.

Discrepancies were observed between the relative risk and univariable COX analyses of MPO-ANCA titres at diagnosis < 81.0 IU/mL for ESKD progression during follow-up. We inferred that the relatively late onset of ESKD progression during the follow-up period may be the cause of this discrepancy. To clarify this, we conducted Kaplan–Meier survival analysis and found that patients with MPO-ANCA titre at diagnosis ≤ 81.0 IU/mL exhibited a pattern of decreased cumulative ESKD-free survival rate compared to those with MPO-ANCA titre at diagnosis > 81.0 IU/mL; however, it was not statistically significant due to a late decrease pattern (Figure S1), as expected. This result suggests that in patients with lower MPO-ANCA titres but high BVAS at diagnosis, rituximab significantly delayed the timing of ESKD progression during the follow-up period.

This study has the strength in that this is the first to demonstrate that lower MPO-ANCA titres at diagnosis is associated with a higher frequency of ESKD progression in rituximab-treated MPA patients. This study also has several limitations. Because of the small number of patients, it was difficult to generalise the results of this study and immediately apply them to patients newly diagnosed with MPA in real clinical settings. Owing to the retrospective study design, it was impossible to strictly control confounding factors affecting ESKD progression during the period between MPA diagnosis and the last visit or the development of ESKD as well. Also, due to this limitation, we could not perform further analyses including diverse comorbidities and paraclinical data affecting progression to ESKD in patients with AAV. Additionally, the detailed histological descriptions of ANCA-associated GN, such as the class of GN or degree of kidney damage from biopsy results, which would have helped to verify the clinical usefulness of initial MPO-ANCA titres, could not be obtained. The absence of a validation cohort was another limitation. Nonetheless, we believe that this study has clinical significance as it is the first pilot study to investigate the correlation between initial MPO-ANCA titres and future ESKD progression among MPA patients in the rituximab treatment setting. We also believe that a prospective future study with more patients will improve these limitations and further provide more reliable and dynamic information on the effect of MPO-ANCA titres at diagnosis on ESKD progression in rituximab-treated MPA patients.

## 5. Conclusions

This is the first study to demonstrate that lower MPO-ANCA titres at diagnosis might be associated with a higher frequency of ESKD progression during follow-up in rituximab-treated MPA patients. We suggest that we should pay more attention to determining induction therapeutic regimens among MPA patients with lower MPO-ANCA titres based on the cut-off of MPO-ANCA titres for ESKD calculated in each cohort. However, at the same time, we should also remember that MPO-ANCA titres at diagnosis should not be an absolute criterion for selecting induction therapeutic regimens.

## Figures and Tables

**Table 1:** Characteristics of rituximab-treated MPA patients with MPO-ANCA (or P-ANCA) and renal vasculitis at diagnosis (N = 34).

Variables	Values
At the time of MPA diagnosis	
Demographic data	
Age (years)	59.0 (50.8–71.3)
Male sex (N, (%))	10 (29.4)
Female sex (N, (%))	24 (70.6)
BMI (kg/m^2^)	21.9 (20.2–25.3)
Ex-smoker (N, (%))	1 (2.9)
ANCA type and positivity (N, (%))	
MPO-ANCA (or P-ANCA) positivity	34 (100)
MPO-ANCA titre (IU/mL)	104.0 (30.8–134.0)
PR3-ANCA (or C-ANCA) positivity	1 (2.9)
AAV-specific indices	
BVAS	15.5 (11.0–19.5)
FFS	2.0 (1.0–2.0)
Acute-phase reactants	
ESR (mm/h)	80.0 (42.0–107.0)
CRP (mg/L)	9.3 (1.5–62.7)
Laboratory results	
White blood cell count (/mm^3^)	7510.0 (5807.5–11,387.5)
Haemoglobin (g/dL)	10.5 (8.8–12.1)
Platelet count (×1000/mm^3^)	296.0 (234.0–372.0)
Fasting glucose (mg/dL)	95.5 (89.0–131.3)
Blood urea nitrogen (mg/dL)	22.2 (17.1–38.3)
Serum creatinine (mg/dL)	1.4 (0.8–2.5)
Serum total protein (g/dL)	6.4 (6.0–7.0)
Serum albumin (g/dL)	3.4 (2.9–4.1)
Random urine protein/creatinine ratio	1.1 (0.8–1.6)
Autoantibodies (N, (%))	
Antinuclear antibody	6 (17.6)
Anti-DNA	0 (0)
Anti-RNP	0 (0)
Anti-Sm	0 (0)
Anti-Ro	2 (5.9)
Anti-La	1 (2.9)
Anti-Scl70	0 (0)
Anti-centromere	0 (0)
Anti-GBM	0 (0)
Comorbidities (N, (%))	
T2DM	5 (14.7)
Hypertension	15 (44.1)
Dyslipidaemia	7 (20.6)
During the follow-up duration	
ESKD (N, (%))	7 (20.6)
Follow-up duration based on ESKD (months)	39.5 (18.3–76.3)
Medications administered	
Rituximab	34 (100)
Glucocorticoids	34 (100)
Mycophenolate mofetil	19 (55.9)
Azathioprine	22 (64.7)
Tacrolimus	8 (23.5)
Methotrexate	5 (14.7)

Values are expressed as a median (25–75 percentile) or N (%). MPA: microscopic polyangiitis; MPO: myeloperoxidase; ANCA: antineutrophil cytoplasmic antibody; P: perinuclear; BMI: body mass index; PR3: proteinase 3; C: cytoplasmic; BVAS: the Birmingham vasculitis activity score; FFS: the five-factor score; ESR: erythrocyte sedimentation rate; CRP: C-reactive protein; DNA: deoxyribonucleic acid; RNP: ribonuclear protein; GBM: glomerular basement membrane; T2DM: type 2 diabetes mellitus; ESKD: end-stage kidney disease.

## Data Availability

The original contributions presented in this study are included in the article/Supplementary Material. Further inquiries can be directed to the corresponding author.

## Supplementary Materials

The following supporting information can be downloaded at: https://www.mdpi.com/article/10.3390/medicina61111892/s1, Table S1: Correlation of MPO-ANCA titre with cross-sectional BVAS, FFS, and continuous laboratory results. Table S2: Cox hazards model analyses of MPO-ANCA titre ≤ 81.0 IU/mL and variables at diagnosis for ESKD progression in RTX-treated MPA patients. Figure S1: Comparison of the cumulative ESKD-free survival rates.

## Abbreviations

The following abbreviations are used in this manuscript:

AAV	antineutrophil cytoplasmic antibody-associated vasculitis
ANCA	antineutrophil cytoplasmic antibody
AUC	area under the curve
BMI	body mass index
BVAS	the Birmingham vasculitis activity score
C	cytoplasmic
CI	confidence interval
ESKD	end-stage kidney disease
ESR	erythrocyte sedimentation rate
FFS	the five-factor score
GN	glomerulonephritis
HR	hazard ratio
IRB	institutional review board
MPA	microscopic polyangiitis
MPO	myeloperoxidase
P	perinuclear
PR3	proteinase 3
ROC	receiver operating characteristic
RR	relative risk
