# Supplementary material for: Lower Myeloperoxidase-ANCA Titres at Diagnosis Are Associated with End-Stage Kidney Disease Progression During Follow-Up in Rituximab-Treated Patients with Microscopic Polyangiitis
Source: Medicina (Kaunas). 2025 Oct 22;61(11):1892. doi: 10.3390/medicina61111892 (PMC12654435; doi:10.3390/medicina61111892)
Supplement: Supplementary file 1 [file medicina-61-01892-s001.zip › Supplementary Table S1 (1stREVISION).pdf]

Table S1. Correlation of MPO-ANCA titre with cross-sectional BVAS, FFS, and continuous laboratory results

Variables	Correlation coefficient (r)	P-value
BVAS	0.163	0.358
FFS	0.101	0.569
ESR (mm/hr)	0.340	0.053
CRP (mg/L)	0.182	0.303
White blood cell count (/mm^3)	0.342	0.048
Haemoglobin (g/dL)	−0.232	0.187
Platelet count (× 1000/mm^3)	0.283	0.105
Fasting glucose (mg/dL)	0.127	0.473
Blood urea nitrogen (mg/dL)	0.156	0.378
Serum creatinine (mg/dL)	0.218	0.215
Serum total protein (g/dL)	0.169	0.340
Serum albumin (g/dL)	−0.233	0.185
Random urine protein/creatinine ratio	0.273	0.118

MPO: myeloperoxidase; ANCA: antineutrophil cytoplasmic antibody; BVAS: the Birmingham vasculitis activity score; FFS: the five-factor score; ESR: erythrocyte sedimentation rate; CRP: C-reactive protein.
