# Supplementary material for: Lower Myeloperoxidase-ANCA Titres at Diagnosis Are Associated with End-Stage Kidney Disease Progression During Follow-Up in Rituximab-Treated Patients with Microscopic Polyangiitis
Source: Medicina (Kaunas). 2025 Oct 22;61(11):1892. doi: 10.3390/medicina61111892 (PMC12654435; doi:10.3390/medicina61111892)
Supplement: Supplementary file 1 [file medicina-61-01892-s001.zip › Supplementary Table S2 (1stREVISION).pdf]

Table S2. Cox hazards model analyses of MPO-ANCA titre ≤81.0 IU/mL and variables at diagnosis for ESKD progression in RTX-treated MPA patients

Variables	Univariable			Multivariable		
	HR	95% CI	P value	HR	95% CI	P value
Age (years)	0.966	0.913, 1.023	0.239			
Male sex (N, (%))	0.028	0.000, 111.231	0.399			
BMI (kg/m^2)	0.862	0.634, 1.172	0.344			
Ex-smoker (N, (%))	0.047	0.000, 1.531E+14	0.867			
BVAS	1.074	0.955, 1.207				
FFS	1.768	0.758, 4.120	0.187			
ESR (mm/hr)	1.001	0.978, 1.024	0.946			
CRP (mg/L)	1.002	0.991, 1.014	0.687			
White blood cell count (/mm^3)	1.000	1.000, 1.000	0.774			
Haemoglobin (g/dL)	0.931	0.638, 1.306	0.618			
Platelet count (× 1000/mm^3)	0.997	0.987, 1.008	0.588			
Fasting glucose (mg/dL)	1.011	0.998, 1.023	0.088	1.015	1.000, 1.031	0.051
Blood urea nitrogen (mg/dL)	1.050	1.000, 1.103	0.049	0.997	0.895, 1.111	0.961
Serum creatinine (mg/dL)	2.069	1.115, 3.839	0.021	3.774	1.034, 13.782	0.044
Serum total protein (g/dL)	0.880	0.304, 2.550	0.815			
Serum albumin (g/dL)	0.759	0.188, 3.064	0.699			
T2DM	3.025	0.308, 29.669	0.342			
Hypertension	1.228	0.243, 6.191	0.804			
Dyslipidaemia	3.635	0.512, 25.815	0.197			
MPO-ANCA titre ≤81.0 IU/mL	4.414	0.469, 36.587	0.201	13.044	0.791, 215.082	0.072

MPO: myeloperoxidase; ANCA: antineutrophil cytoplasmic antibody; ESKD: end-stage kidney disease; RTX: rituximab; MPA: microscopic polyangiitis; HR: hazards ratio; CI: confidence interval; BMI: body mass index; BVAS: Birmingham vasculitis activity score; FFS: five-factor score; ESR: erythrocyte sedimentation rate; CRP: C-reactive protein; T2DM: type 2 diabetes mellitus.
