# Supplementary figures and images for: Lower Myeloperoxidase-ANCA Titres at Diagnosis Are Associated with End-Stage Kidney Disease Progression During Follow-Up in Rituximab-Treated Patients with Microscopic Polyangiitis
Source: Medicina (Kaunas). 2025 Oct 22;61(11):1892. doi: 10.3390/medicina61111892 (PMC12654435; doi:10.3390/medicina61111892)

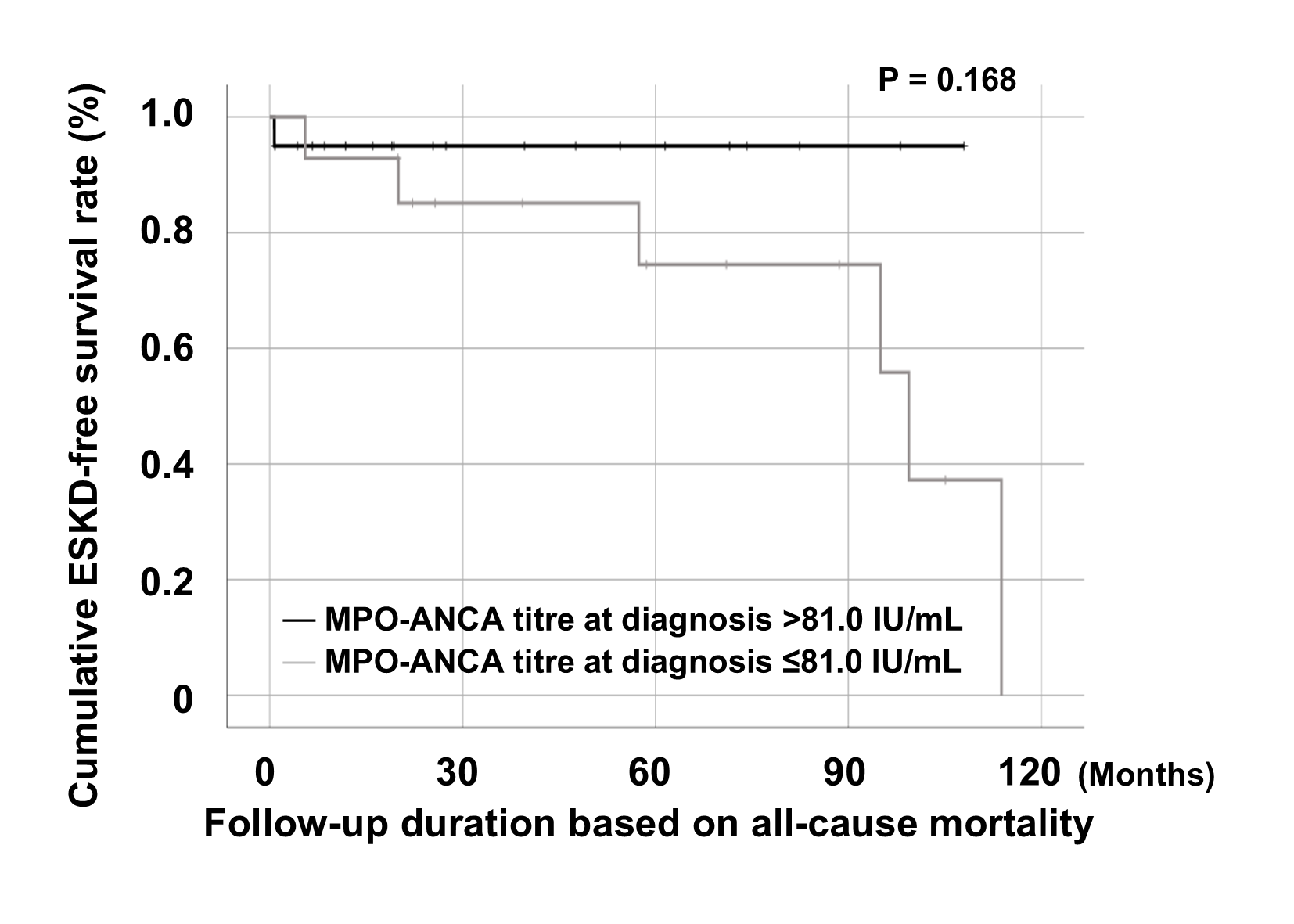

Supplement: Supplementary file 1 [file medicina-61-01892-s001.zip › Supplementary Figure S1 (1stREVISION).tif]
